# Development of a Deep Learning Algorithm for Periapical Disease Detection in Dental Radiographs

**DOI:** 10.3390/diagnostics10060430

**Published:** 2020-06-24

**Authors:** Michael G. Endres, Florian Hillen, Marios Salloumis, Ahmad R. Sedaghat, Stefan M. Niehues, Olivia Quatela, Henning Hanken, Ralf Smeets, Benedicta Beck-Broichsitter, Carsten Rendenbach, Karim Lakhani, Max Heiland, Robert A. Gaudin

**Affiliations:** 1 Laboratory for Innovation Science, Harvard University, 175 N. Harvard Street, Suite 1350, Boston, MA 02134, USA; m.g.endres@gmail.com; 2 Institute for Data, Systems and Society, Massachusetts Institute of Technology, 50 Ames St, Cambridge, MA 02142, USA; florian.hillen@videahealth.io; 3 Department of Oral- and Maxillofacial Surgery, Charité-Universitätsmedizin Berlin, Corporate Member of Freie Universität Berlin, Humboldt-Universität zu Berlin, and Berlin Institute of Health, Hindenburgdamm 30, 12203 Berlin, Germany; marios.salloumis@charite.de (M.S.); carsten.rendenbach@charite.de (C.R.); benedicta.beck-broichsitter@charite.de (B.B.-B.); max.heiland@charite.de (M.H.); 4 Department of Otolaryngology—Head and Neck Surgery, University of Cincinnati College of Medicine, Medical Sciences Building Room 6410, 231 Albert Sabin Way, Cincinnati, OH 45267, USA; ahmad.sedaghat@uc.edu; 5 Department of Radiology, Charité-Universitätsmedizin Berlin, Corporate Member of Freie Universität Berlin, Humboldt-Universität zu Berlin, and Berlin Institute of Health, Hindenburgdamm 30, 12203 Berlin, Germany; stefan.niehues@charite.de; 6 Department of Oral- and Maxillofacial Surgery, Universitätsklinikum Hamburg, Eppendorf, Maritnistraße 52, 20246 Hamburg, Germany; oquatela@u.rochester.edu (O.Q.); r.smeets@uke.de (R.S.); h.hanken@uke.de (H.H.); 7 Technology and Operations Management Unit, Harvard Business School, Wyss House, Boston, MA 02163, USA; klakhani@hbs.edu

**Keywords:** artificial intelligence, diagnosis, computer-assisted, image interpretation, computer-assisted, machine learning, radiography, panoramic radiograph

## Abstract

Periapical radiolucencies, which can be detected on panoramic radiographs, are one of the most common radiographic findings in dentistry and have a differential diagnosis including infections, granuloma, cysts and tumors. In this study, we seek to investigate the ability with which 24 oral and maxillofacial (OMF) surgeons assess the presence of periapical lucencies on panoramic radiographs, and we compare these findings to the performance of a predictive deep learning algorithm that we have developed using a curated data set of 2902 de-identified panoramic radiographs. The mean diagnostic positive predictive value (PPV) of OMF surgeons based on their assessment of panoramic radiographic images was 0.69 (±0.13), indicating that dentists on average falsely diagnose 31% of cases as radiolucencies. However, the mean diagnostic true positive rate (TPR) was 0.51 (±0.14), indicating that on average 49% of all radiolucencies were missed. We demonstrate that the deep learning algorithm achieves a better performance than 14 of 24 OMF surgeons within the cohort, exhibiting an average precision of 0.60 (±0.04), and an F_1_ score of 0.58 (±0.04) corresponding to a PPV of 0.67 (±0.05) and TPR of 0.51 (±0.05). The algorithm, trained on limited data and evaluated on clinically validated ground truth, has potential to assist OMF surgeons in detecting periapical lucencies on panoramic radiographs.

## 1. Introduction

Panoramic radiographs are a common diagnostic tool and a standard imaging modality that is frequently employed in routine clinical practice by dentists and oral and maxillofacial (OMF) surgeons [1,2,3]. Although assessment of panoramic radiographs may be contracted to radiologists in certain circumstances, in many clinical practices, OMF surgeons often read their own panoramic radiographs. Previous research has shown that a physician’s training plays an integral role in correctly interpreting medical imaging [4]. In dentistry fields specifically, the agreement rate (a proxy for their diagnostic performance) of dental professionals’ assessments of radiographic images seems to vary in part due to individual knowledge, skills and biases [5,6]. The variability in dental professionals’ abilities to read panoramic radiographs opens the door for misdiagnosis or mistreatment [7,8]. For example, recent research has shown that the rate of misdiagnosis by dentists in determining the depth of caries in a conventional radiograph was as high as 40 percent, and in 20 percent of cases, teeth were misdiagnosed as diseased [9,10].

In medicine, much recent research has focused on developing diagnostic and therapeutic artificial intelligence (AI) tools to support the clinical decision-making process [11,12,13,14]. So far, AI has been introduced and used in many clinical specialties such as radiology [12,15,16], pathology [17,18,19], dermatology [20] and ophthalmology [21,22] to aid with the detection of disease and the subsequent recommendation of treatment options. AI algorithms have also been developed for segmenting medical images for therapeutic tasks, such as tumor delineation in the head and neck for targeting by radiation therapy [23]. Previous work in computer-aided diagnostics in dentistry and OMF surgery is limited. Prior studies focused on caries detection in bitewing radiographic images as well as tooth segmentation and for orthodontic calculations [24,25,26,27]. The only Food and Drug Administration (FDA) approved tool to date, the Logicon caries detector, was introduced in 1998, and is intended only for detecting and precisely diagnosing the depth of inter-proximal caries lesions [28].

Detection of radiolucencies in a panoramic radiograph is a common task for OMF surgeons [29]. In fact, the prevalence of periapical radiolucencies in radiographic images obtained in dental outpatient departments has been reported to be approximately 9–10% [29,30,31]. The presence of periapical radiolucencies may reflect some common or serious dental diseases including infection (accounting for approximately 55–70% of radiolucencies), cysts (25–40% of radiolucencies), granulomas (1–2% of radiolucencies) and tumors [29,30,31]. Delayed diagnoses of these radiolucent periapical alterations can lead to spread of disease to surrounding tissues, complications and patient morbidity [32]. Although many dentists and OMF surgeons read their own panoramic radiographs, there has been little research conducted to study their accuracy in identifying the common radiolucent periapical alterations. In this study, we investigated the detection of periapical radiolucencies on panoramic radiographs. We studied the ability with which OMF surgeons identified the presence of periapical radiolucencies in panoramic radiographs. Additionally, we used deep learning to develop an image analysis algorithm for the detection of periapical radiolucencies on panoramic radiographs that could serve as an aid in clinical practice, and compared its performance with that of OMF surgeons.

## 2. Materials and Methods

Images for this study were obtained from the outpatient clinic at the Department of Oral and Maxillofacial Surgery, Charité, Berlin. In the Department of Oral and Maxillofacial Surgery, Charité, Berlin, panoramic radiographs are used as the standard imaging modality due to its overall good diagnostic discriminatory ability. Furthermore, this modality allows an overview by assessing the entire dentition plus surrounding bony structures, while using low doses of radiation [33,34,35]. Nevertheless, the overall standard in endodontic radiography for the detection of radiolucent periapical alterations, especially for the detection of apical periodontitis, is periapical radiography [33].

The use of the images and the participation of OMF surgeons in this study is approved by the institutional review board at Harvard University (board reference number: IRB17-0456; date of approval: 01 May 2018) and Charité, Berlin (board reference number: EA2/030/18; date of approval: 15 March 2018). Written informed consent for the study was obtained from all participating OMF surgeons. All methods and experiments were carried out in accordance with relevant guidelines and regulations (Declaration of Helsinki). The annotation of all panoramic radiographs took place in standardized radiology reading rooms including a clinical radiology monitor connected to the hospital’s information technology system. All participating OMF surgeons annotated the images on a web-based application, which was developed for this study.

### 2.1. Assessing the Reliability of OMF Surgeons’ Diagnoses of Periapical Radiolucenies in Panoramic Radiographs

For the evaluation of the reliability of diagnosis of periapical radiolucencies in panoramic radiographs by OMF surgeons in routine clinical practice, 24 OMF surgeons were recruited (eighteen from the Department of Oral and Maxillofacial Surgery, Charité, Berlin, three from the Department of Oral and Maxillofacial Surgery, University Clinic Hamburg, Eppendorf, and three from private practices for OMF surgery). These OMF surgeons represented a random sample comprising 13 residents and 11 attending physicians (6 female and 18 male).

OMF surgeons were instructed to annotate 102 de-identified panoramic radiographs for clinically relevant periapical radiolucencies (Table 1). The reference standard data was collected by a single OMF surgeon with 7 years of experience who treated all 102 unique patients using the following procedure. First, a panoramic radiograph was taken of the patient and evaluated; all detected radiolucencies were subsequently recorded. Second, every tooth of the patient was tested for clinically relevant periapical diseases (e.g., abscess) using pulp vitality testing through thermal and percussion testing—a gold standard for clinically validating periapical diseases [7]. In general, teeth with periapical disease do not show any response to the different testing methods compared to healthy teeth, due to the loss of vitality. Consequently, the OMF surgeon has additional clues as to whether the periapical radiolucency is an artifact or indeed due to disease, compared to relying solely on the radiograph. If a radiolucency had been missed by the OMF surgeon’s reading but periapical disease was subsequently detected by the clinical test, the radiographic image was then assessed a second time to determine whether the radiolucent periapical alteration was visible and then recorded.

### 2.2. Development of a Deep Learning Algorithm for the Automated Detection of Periapical Radiolucencies in Panoramic Radiographs

We developed our model using a supervised learning approach, whereby the functional relationship between an input (i.e., radiographic images) and output (i.e., a list of detected radiolucent periapical alteration locations, and corresponding confidence scores) is “learned” by example. The task generally requires multiple labeled data sets: a data set used for the purpose of training the model, a validation data set used to determine whether or not the model is over-fitting the training set and for the purpose of selecting the best among several candidate models, and a testing data set used for final evaluation of the selected model. We assessed our model by comparing its performance on the same 102 images annotated by the 24 OMF surgeons described in Section 1 of Methods and Materials.

### 2.3. Radiographic Images and Labelling for Model Training

The training data set, comprising 3240 radiographic images, was labeled by four OMF surgeons from the Department of Oral and Maxillofacial Surgery, Charité, Berlin, from the same outpatient department (experience ranging from 5 to 20 years) who visually assessed the images, absent of any additional clinical information, and subsequently produced contour labels around any visible and treatable periapical radiolucencies that they had identified (Table 1). Of note, physicians starting the OMF surgery residency program in Germany have already had at least two years experience reading dental radiographs and treating patients due to the program in dental school. Furthermore, in Germany, dentomaxillofacial radiology training is part of the OMF surgery residency program. No single subspecialty of OMF radiology exists in Germany.

**Figure 1:**
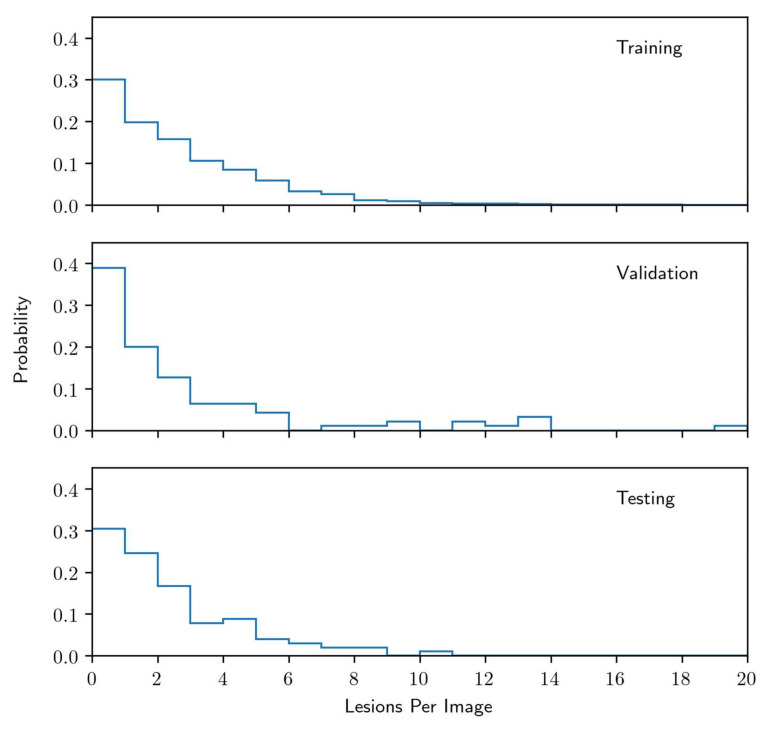
Distribution of radiolucent periapical alterations per image for the training data set, the validation data set, and the testing data set.

Among the 3240 images assessed, 338 were excluded from the training data set. The exclusion criteria included inappropriate anatomy coverage due to poor positioning or artifacts, inferior density and poor contrast between enamel and dentin, as well as inferior density and poor contrast of the tooth itself with the bone surrounding it. These criteria comply with standards stated in the literature [5,36]. The radiolucent periapical alteration distribution of the remaining 2902 labeled images is shown in Figure 1, and among the retained images, 872 were assessed as being free of visible radiolucencies.

### 2.4. Reference Standard for Model Selection and Evaluation

A separate set of 197 panoramic radiographic images and associated diagnoses were collected from the Department of Oral and Maxillofacial Surgery, Charité, Berlin. This data represented a reference standard for both model selection and final evaluation purposes. The images and labels were collected and produced by a single OMF surgeon with seven years of experience. The diagnoses were made by the OMF surgeon, who took and assessed the radiographic image of each patient, subsequently clinically tested each tooth within the patient’s jaws using percussion and thermal vitality tests. The data set was split into two disjointed subsets at the patient level: a 95 image validation set (used for model selection) and a 102 image test set (same as described in Section 2.1 of Methods and Materials) which was used for final evaluation of our trained model. Associated radiolucent periapical alteration distributions for these sets, along with the training data set, are shown in Figure 1.

### 2.5. Benchmarks for Model Comparison

The model performance was compared against a benchmark of 24 OMF surgeons. The protocols for diagnosing the images were identical to those provided to the OMF surgeon who labeled the training data sets, however, the OMF surgeons were asked to produce a single point at the center of each radiolucent periapical alteration as opposed to a tight contour.

### 2.6. Model

**Figure 2:**
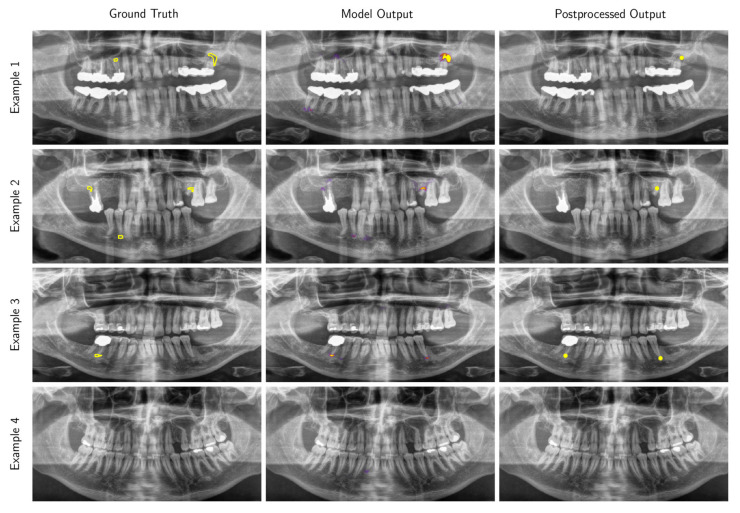
Examples of panoramic radiographic images (preprocessed for model input) selected from the test data set with overlays of the ground truth contours (Ground Truth), the intensity map output produced by our model (Model Output) and locations produced by our post-processing procedure (Postprocessed Output). Only predictions with a confidence score greater than 0.25 are displayed (this threshold was selected to maximize the F_1_ score on the validation data set). Higher resolution versions of these images are provided in Figure A1, Figure A2, Figure A3 and Figure A4.

**Figure A1:**
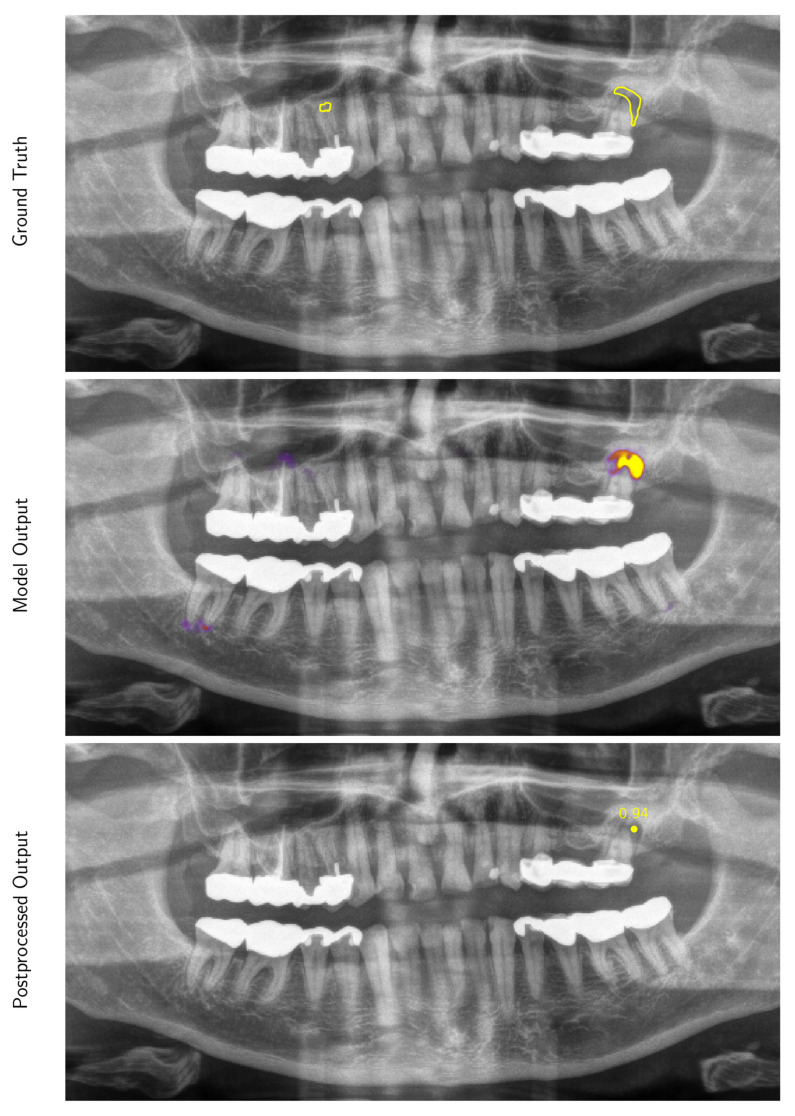
Example 1 of a panoramic radiographic image (preprocessed for model input) selected from the test data set with overlays of the ground truth contours (Ground Truth), the intensity map output produced by our model (Model Output) and locations produced by our post-processing procedure (Postprocessed Output). Only predictions with a confidence score greater than 0.25 are displayed as an example (this threshold was selected to maximize the F_1_ score on the validation data set).

**Figure A2:**
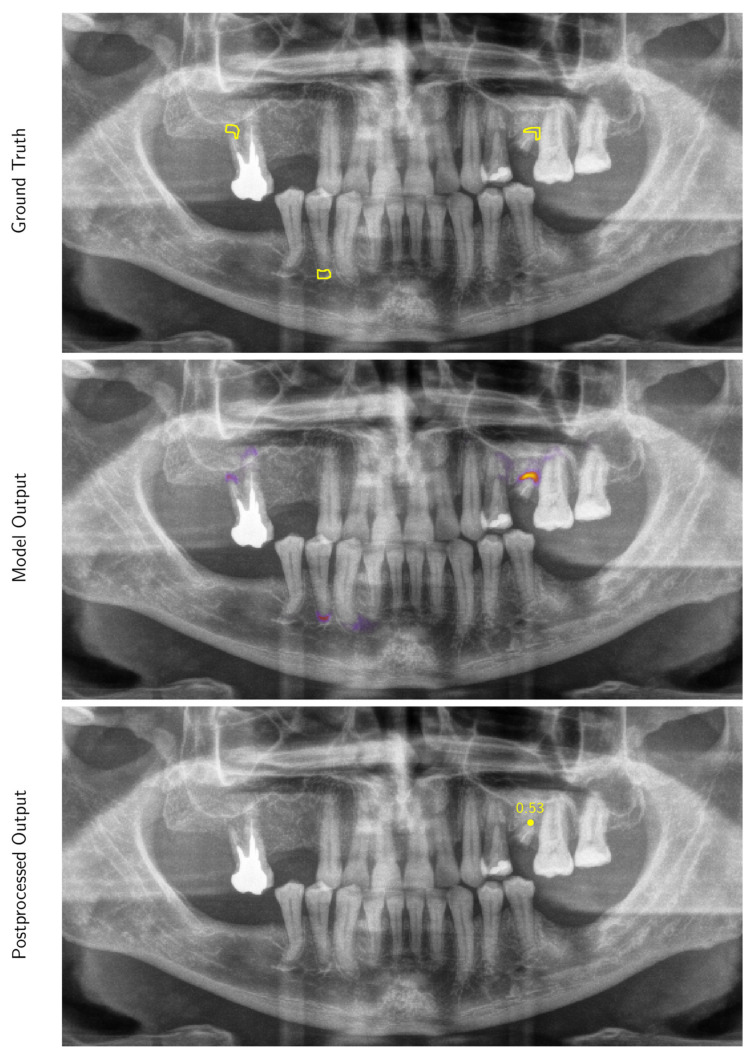
Example 2; see Figure A1 for details.

**Figure A3:**
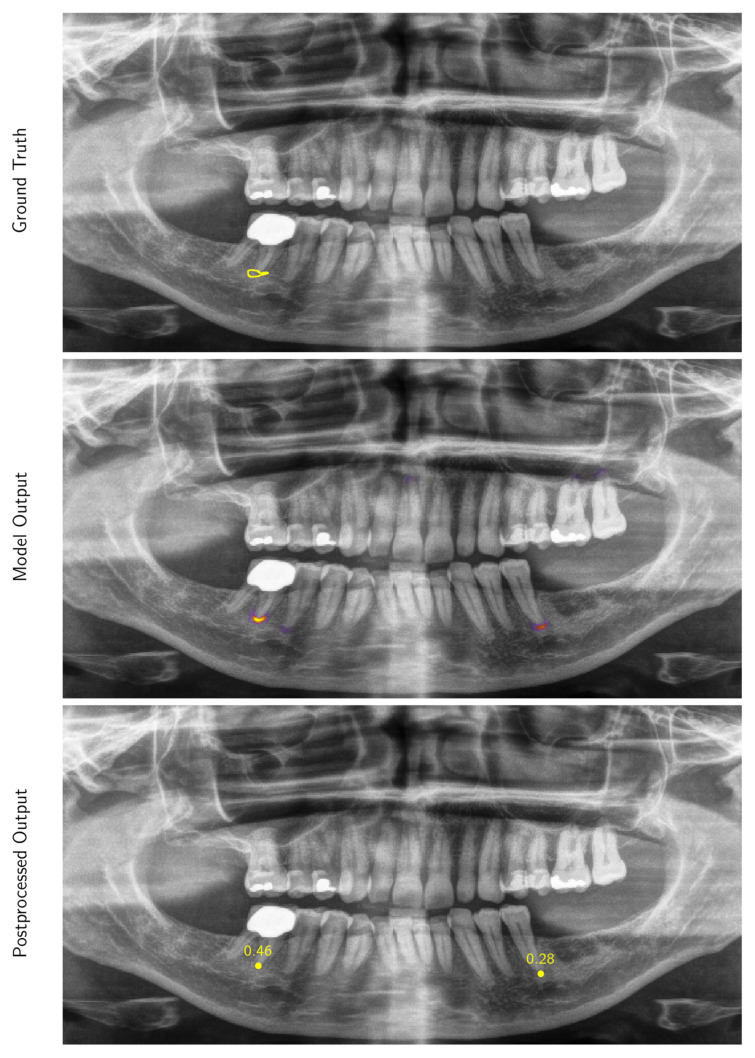
Example 3; see Figure A1 for details.

**Figure A4:**
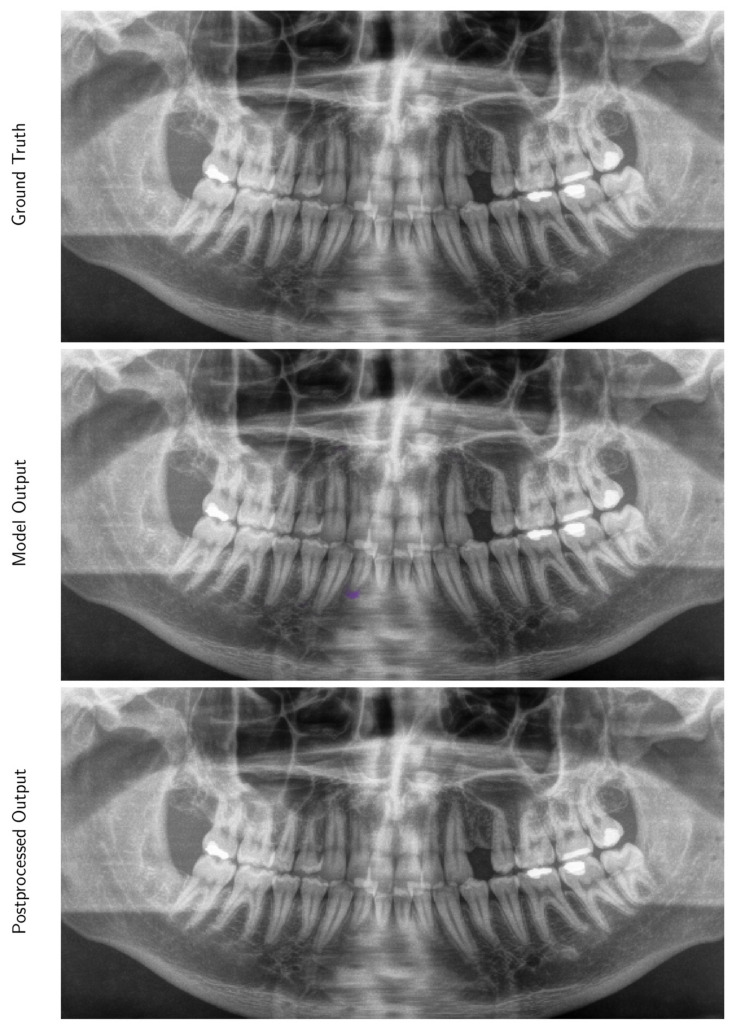
Example 4; see Figure A1 for details.

**Figure A5:**
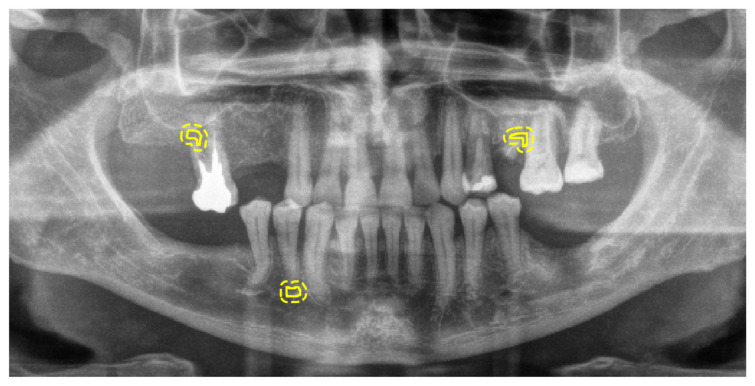
Example of a preprocessed panoramic radiographic image, selected from the test data set, with overlays of the ground truth contours (solid) and four-pixel error tolerance regions (dashed).

We framed the radiolucent periapical alteration detection task as a dense classification problem, whereby each pixel in an input radiographic image is determined to be either containing a radiolucent periapical alteration or not (see Appendix A for full details). The model was based on a deep convolutional neural network for image segmentation [37], which output an intensity map indicating regions of high or low confidence of containing a radiolucent periapical alteration. These intensity maps were subsequently postprocessed to yield a list of location points of a radiolucent periapical alteration within the image, and associated confidence scores on the interval (0,1) (Figure 2 and Figure A1, Figure A2, Figure A3, Figure A4 and Figure A5).

### 2.7. Evaluation Metrics

The performance of our model was assessed in terms of positive predictive value (commonly referred to as “precision”), PPV = N_TP_/(N_TP_ + N_FP_), true positive rate (commonly referred to as “sensitivity” or “recall”), TPR = N_TP_/(N_TP_ + N_FN_), and F_1_ score (a commonly used performance metric in machine learning, defined as the harmonic average of the PPV and TPR), where N_TP_ is the true positive (TP) count, N_FP_ is the false positive (FP) count and N_FN_ is the false negative (FN) count for predictions on the entire data set considered (see Appendix A for full details). The model was also assessed using average precision (AP), defined as the area under the PPV-TPR curve based on Riemann summation. Performance metrics were determined as a function of a confidence threshold, treating locations with confidence scores greater than the threshold as positive predictions.

### 2.8. Evaluation of Correlations between Model and OMF Surgeons’ Performance

In addition to traditional evaluation metrics and benchmark comparisons, we studied the relationship between our model confidence scores and those inferred from the cohort of 24 OMF surgeons. The locations identified as a radiolucent periapical alteration by the 24 OMF surgeons in the testing data set were manually clustered by an OMF surgeon based on either the radiolucent periapical alteration locations indicated by the reference standard or by root location in the case of negative condition instances. A contour region was then produced around each cluster and a cohort confidence score was assigned to each region based on the proportion of OMF surgeons that found the region to be a radiolucent periapical alteration. Within each region, we additionally determined a model confidence score based on the predictions of the model for the purpose of comparison. We then used Spearman’s rank correlation coefficient to assess the monotonic relationship between the model and the cohort confidence scores.

## 3. Results

### 3.1. The Reliability of OMF Surgeons’ Diagnoses of Periapical Radiolucenies in Panoramic Radiographs

In total, 2448 images (102 images per OMF surgeon, for 24 OMF surgeons) were annotated in this study. The results from this analysis (Table 2) demonstrate that for the task of detecting radiolucencies using panoramic radiographs, OMF surgeons had a mean PPV of 0.69 (±0.13), indicating that on average 31% of cases were falsely diagnosed as positive (type I error). The mean TPR of all OMF surgeons was 0.51 (±0.14), indicating that on average 49% of all radiolucencies were missed (type II error). The performance of the OMF surgeons was significantly lower in terms of TPR compared to PPV based on a Wilcoxon signed-rank test (*p* = 0.003). Based on the performance distribution (Table 2), the probability that the OMF surgeons had a PPV greater than 0.5 was 96 (±4)% whereas the probability that they had a TPR greater than 0.5 was only 50 (±10)%, where the values in parentheses represent 1σ statistical uncertainties in the estimates.

**Figure 3:**
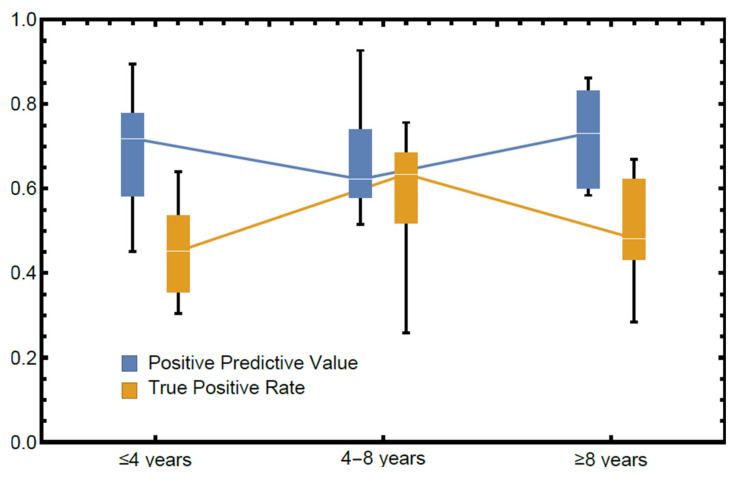
Performance stratified by self-reported years of experience in diagnosing panoramic radiographs (lines indicate median, boxes span the first and third quartiles and fences span the total range). Groups contain 9 (≤4 years), 6 (4–8 years), and 9 (≥8 years) OMF surgeons, respectively.

The relationship between experience in diagnosing periapical radiolucencies and performance can be seen in Figure 3, where OMF surgeons have been divided into three approximately equal-in-size groups (≤4 years, 4–8 years, and ≥8 years). The mean performance within the plot suggests that there is no significant effect on either PPV or TPR due to experience. 

### 3.2. Performance of the Deep Learning Algorithm

**Figure 4:**
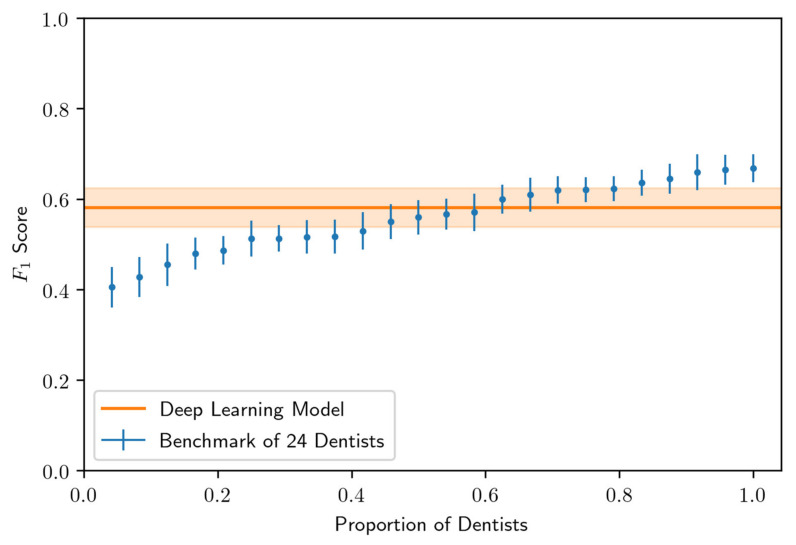
Comparison of 24 OMF surgeon and model predictions in terms of F_1_ score on the testing data set. The model threshold was chosen so that the F_1_ score was maximized on the validation data set. Standard errors (whiskers and uncertainty bands) were computed via a jackknife analysis.

A comparison of the model performance and 24 OMF surgeons in terms of F_1_ score is shown in Figure 4. In terms of this metric, we found that the model outperforms 14 of the 24 OMF surgeons (58% of the OMF surgeons) at the best performing confidence threshold of 0.25, determined using the validation data set. The model yielded an AP of 0.60 (±0.04) and F_1_ score of 0.58 (±0.04) corresponding to a PPV of 0.67 (±0.05) and TPR of 0.51 (±0.05), where standard errors in parenthesis were determined using a jackknife analysis. These performance results are consistent with the mean PPV and TPR measured for the cohort of 24 OMF surgeons.

**Figure 5:**
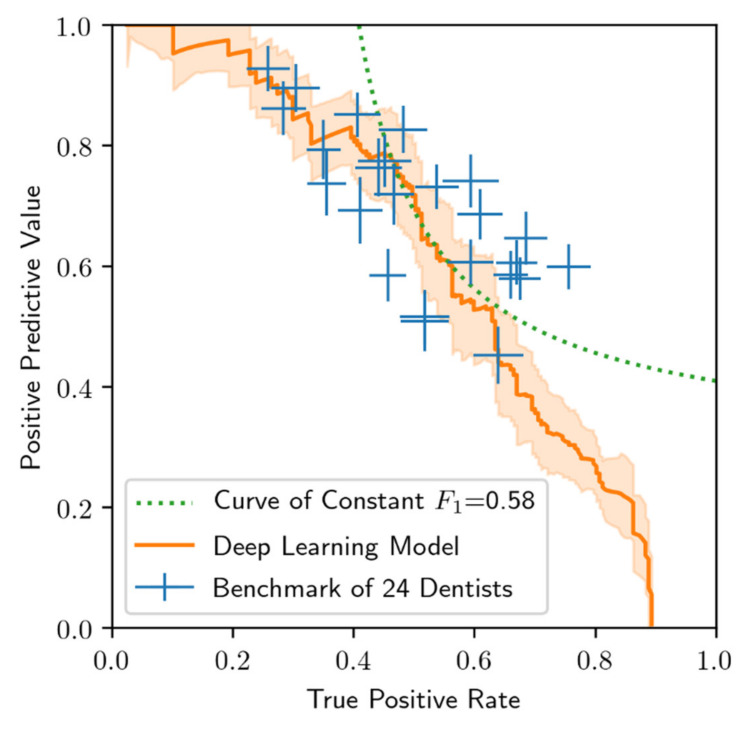
Comparison of 24 OMF surgeon and model performance on the test data set. Standard errors (whiskers), computed via a jackknife analysis. The curve of constant F_1_ score equal to 0.58 shown is used to compare performance results in Figure 3.

The model performance (PPV as a function of TPR, also commonly known as a precision-recall curve) is shown in Figure 5, and compared with the benchmark performance of the 24 OMF surgeons. The curve and standard error band is determined by parametrically mapping out the PPV and TPR as a function of the confidence threshold. In particular, as the confidence threshold vanishes, the PPV vanishes but the TPR attains a maximum value of approximately 0.9. On the other hand, as the confidence threshold approaches unity, the PPV approaches unity, but at the cost of diminished TPR. The optimal threshold is user-defined, and is dependent on external factors such as the relative health risks and costs associated with having an excess of FP cases versus an excess of FN cases. Of note, the model exceeded the performance of approximately half of the 24 OMF surgeons (i.e., those who demonstrate PPV and TPR below and to the left of the model curve in Figure 5) in that for these OMF surgeons there always exists a confidence threshold whereby the model exhibits better performance in both PPV and TPR.

**Figure 6:**
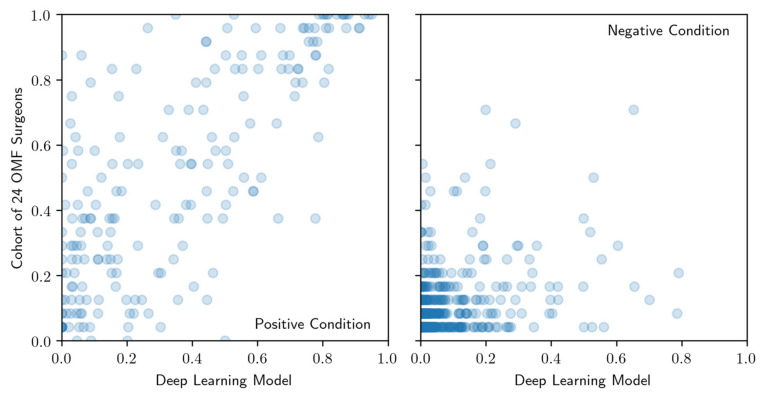
Comparison of confidence score rankings for positive condition cases (left) and negative condition cases (right) produced by the model (axis labeled Deep Learning Model) and cohort of OMF surgeons (axis labeled Cohort of OMF surgeons). Regions of interest that are scored most (least) likely to be a radiolucent periapical alteration have highest (lowest) rank.

The correlation between the model and cohort of OMF surgeons confidence score ranking is shown separately in Figure 6 for the positive condition cases (presence of a periapical radiolucency) and the negative condition cases (absence of a periapical radiolucency) identified by the cohort of OMF surgeons. For the positive condition cases, the Spearman correlation is 0.72 (*p*-value: < 0.001), whereas for the negative condition cases, the Spearman correlation is 0.34 (*p*-value: < 0.001). The positive correlation indicates that the model confidence scores tend to increase monotonically with the cohort confidence scores. Interestingly, this correlation appears significantly stronger for positive condition cases compared to negative condition cases. The strong correlation in the former suggests that both the model and the cohort of OMF surgeons find the same kinds of radiolucent periapical alterations either easy or difficult to detect. The weaker correlation in the latter, however, suggests greater complementarity between the model and cohort for the negative condition cases.

## 4. Discussion

While advances in digital radiography have been a major focus of medical research in recent years, a similar focus has been lacking in dentistry. Although OMF surgeons are routinely reading panoramic radiographs in practice, our study demonstrates that the ability of OMF surgeons to identify periapical radiolucencies in panoramic radiographs may be limited. Specifically, the results suggest that radiolucent periapical alterations may be missed, leading to poorer patients’ outcomes or in worst case mortality in an emergency setting and in total exposes OMF surgeons to significant liability. Based on these findings, we developed a machine learning algorithm for identification of periapical radiolucencies which not only performed better than half of experienced OMF surgeons compared against by some metrics, but may serve as a complementary tool in making these diagnoses as well as serving as the foundation for a more comprehensive and fully automated periapical radiolucency detection tool in the future.

Our results closely match a recently published study that reported on an algorithm for detecting apical radiolucencies in panoramic dental radiographs for endodontic treatment [33] with a TPR of 0.65 (±0.12) and PPV of 0.49 (±0.10). This study group chose a different approach by evaluating the algorithm on a dataset labeled based on the interrater agreement of six dentists. The results therefore may be less reliable than our methodology, which includes clinical cross-checking of the labeled radiolucent periapical alterations for establishing the ground truth. Furthermore, in that study [33], the images were labeled by dentists who generally use periapical radiographic images for endodontic treatment rather than panoramic radiographs, which may further limit their reliability. Notably, a final evaluation of model performance on a holdout test data set (i.e., a data set that is untouched until after the process of training, hyperparameter tuning and model selection) had not been performed, thus making their results susceptible to overfitting on the validation data set.

Although our results are promising, there remain several limitations to this study. First, our algorithm was trained on data labeled by OMF surgeons based on readings of radiographs as opposed to clinical testing. As a consequence, our algorithm may reflect the inherent limitations and biases of those OMF surgeons. Such limitations and biases, if learned by our algorithm, would be reflected in a degradation of performance on the testing data set. It is important to note that such issues do not invalidate our study since the test set was labeled based on the outcomes of clinical tests. However, by addressing such issues, better performance may be attained. While it is tempting to assume that a training data set labeled by multiple readers would improve the situation, this may not be the case if the limitations and biases of those readers are correlated. The strong correlation found between the confidence score rankings of our model and that inferred from a cohort of 24 OMF surgeons who read the same radiographs suggests that there may indeed be commonalities between the drivers for misdiagnosis between the model and the cohort, which merits further exploration (e.g., by studying the performance of the model and OMF surgeon cohort on subpopulations, ideally of a much larger test data set). A better understanding of these drivers, whether they be related to image quality, inherent aspects of the radiolucent periapical alterations (e.g., level of progression) or educational differences, may better inform the data collection process for model training. It is important to note, however, that even with clinically or histologically validated training data labels, such issues may persist.

Second, although we evaluated our model using clinically validated labels to establish the ground truth, there remains potential for mislabeling since such clinical tests are subject to misinterpretation and in our case were conducted by one experienced OMF surgeon. This can be controlled, for example, by performing multiple clinical tests of the same patient by multiple OMF surgeons, although this would be a costly endeavor for both the OMF surgeon and the patient. Despite this limitation, we believe using labels based on clinical tests is nevertheless better than common alternatives for labeling, such as inter-observer agreement, which has inherent biases and limitations.

Finally, although we have tested our algorithm on an independent data set of 102 images with clinically validated labels, further tests will be required to demonstrate generalizability of our model to data collected from other sites. The concern here, again, centers on biases that may be learned from training data collected from a single source (for example, if imaging practices differ by institution or if patient populations differ). In future studies, a training set collected from multiple sites would likely lead to greater robustness of the algorithm across sites.

In general, a major challenge for ML applications in radiology remains of how to attain a super-human level of performance. In this work, achieving such levels of performance will require a larger, higher quality labeled training data set. Literature has shown that such performance may be possible by increasing our dataset size 10- to 100-fold and through multiple labeling of the same training data set by different annotators or by acquiring clinically, if possible, even histologically, validated labels for the training data set [38,39]. These strategies, however, would come at significant cost due to the human-expert resources required. It is important to note, however, that histological diagnoses have limitations as well. Although in this case the PPV is expected to be 1.0 (all case instances diagnosed as positive are positive), the TPR will presumably remain less than 1.0, since a dentist/ OMF surgeon must make a decision about whether or not to perform a histological diagnosis. Evidence of a radiolucent periapical alteration would not necessarily lead to extraction of the tooth in order to obtain tissue samples for histological analysis. Without some prompt to take the necessary tests (e.g., due to a missed indication on a radiograph or no reports of pain), the lesion may still be missed. Because of this, we see value in offering an algorithmic solution to enhance the likelihood of drawing attention to potential lesions in a radiograph, prompting the dentist/ OMF surgeon to perform further tests.

The question remains as to why panoramic views were used to diagnose periapical radiolucencies instead of, for example, periapical radiographs in this study. Radiolucent periapical alterations can be detected with several different image modalities, with periapical radiographs being the standard for endodontic radiography. However, this modality displays only one or a few teeth, and when measured against a gold standard (i.e., in cadaver or histological studies) it showed a low discriminatory performance [40]. Cone-beam computed tomography (CBCT) is a 3D image modality that has shown the best discriminatory performance [41]. Nevertheless, it has a limited use due to high costs and associated radiation dose. Panoramic radiographs on the other hand has an overall good diagnostic discriminatory ability and allows the assessment of the entire dentition plus surrounding bony structures, while requiring significantly lower doses of radiation compared to CBCT imaging [33,34,35]. Herein, many general dental practices as well as OMF surgeons choose to use panoramic radiography due to these benefits [33].

Artificial intelligence has the potential to improve clinical outcomes and further raise the value of medical imaging in ways that lie beyond our imaginations. Especially in medical imaging, AI is rapidly moving from an experimental phase to an implementation phase. Given the major advances in image recognition through deep learning, it is tempting to assume that the role of the radiologist will soon diminish. However, this notion disregards the regulatory limitations placed on the use of AI in a clinical setting. For example, the FDA and the European Conformity committee (CE) presently only allow such software as an assistive device. The complex work of radiologists includes many other tasks that require common sense and general intelligence, by integrating medical concepts from different clinical specialties and scientific fields that cannot yet be achieved through AI.

In addition to regulatory barriers, the impact of AI in dentistry and any other specialty will depend on human–machine interactions. Questions remain around how likely an expert would take the suggestion of an algorithm and perform further tests. How does presentation of the AI prediction impact the expert’s response? Would patients trust such algorithms? How do the answers to these questions vary by culture or with time as confidence in AI grows? We do not make any attempt to address such questions in this study, but understanding these issues will be important for the future of AI in medical imaging.

## 5. Conclusions

In this study, we have demonstrated that a deep learning model trained on a curated data set of 2902 de-identified radiographic images, can match the mean diagnostic performance of 24 OMF surgeons in the task of detecting periodical radiolucent alterations. The mean PPV for the OMF surgeons was 0.69 (±0.13), and the mean TPR was 0.51 (±0.14) on a hold-out test data set of 102 radiographs. By comparison, the mean PPV for the model was 0.67 (±0.05) and the mean TPR was 0.51 (±0.05), corresponding to an F_1_ score of 0.58 (±0.04). The AP for the model was 0.60 (±0.14). The rank correlation between model and cohort confidence scores for positive and negative condition cases was 0.72 and 0.34, respectively.

AI is on the verge of becoming a valuable asset to professionals in healthcare. Although further research is needed to address the myriad of remaining open questions; our work provides a promising first step toward realizing an ML-based assistive tool in dentistry that is competitive with OMF surgeons at detecting radiolucent periapical alterations based on visual assessment of radiographs. As the role of AI in healthcare becomes more prominent, we are optimistic that healthcare organizations will adjust their data collection practices to better align with the needs of ML, which will ultimately clear the pathway for online learning (models that continually learn and improve) as well as the development of data-fused models that combine radiographs with other patient data to produce highly reliable diagnoses.

## Figures and Tables

**Table 1:** Detailed description of the lesions considered in the study.

Radiolucent Periapical Alterations	Characteristics [32]
Periapical inflammation/infection	Widened periodontal ligament
Periapical granuloma	Small lucent lesion with undefined borders (<200 mm^3^)
Periapical cysts	Round-shaped and well-defined lesions with sclerotic borders around the tooth root (>200 mm^3^)
Osteomyelitis	Lesion with irregular borders and irregular density, often spread over more than one root
Tumor	Lesion with irregular borders and irregular density, often spread over more than one root

**Table 2:** Performance metrics for each OMF surgeon, based on their assessment of 102 radiographic images (2248 images read in total) and survey response.

Dentist	A	B	C	TPR	PPV
1	≤4	23	8	0.36	0.74
2	≤4	22	11	0.35	0.79
3	≤4	43	2	0.59	0.79
4	≤4	41	8	0.52	0.51
5	≤4	54	10	0.30	0.90
6	≤4	69	19	0.45	0.77
7	≤4	30	2	0.64	0.45
8	≤4	59	10	0.41	0.69
9	≤4	27	8	0.47	0.72
10	4–8	32	2	0.68	0.58
11	4–8	51	4	0.69	0.65
12	4–8	119	0	0.59	0.74
13	4–8	22	0	0.52	0.42
14	4–8	25	8	0.26	0.93
15	4–8	27	8	0.76	0.60
16	≥8	58	8	0.46	0.58
17	≥8	17	7	0.48	0.83
18	≥8	34	10	0.54	0.73
19	≥8	45	14	0.44	0.76
20	≥8	43	12	0.67	0.61
21	≥8	25	9	0.28	0.86
22	≥8	63	5	0.61	0.69
23	≥8	21	1	0.41	0.85
24	≥8	58	9	0.66	0.59
Mean	7.6	42	6.9	0.51	0.69
Median	6.0	38	8.0	0.50	0.71

Column labels: A: Years of experience analyzing panoramic radiographic images (split into three approximately equal sized groups); B: median time spent per image (seconds); C: hours worked prior to task. The 1σ uncertainty in the PPV and TPR of each dentist ranges from 0.03–0.06.

## Appendix A

### Appendix A.1. Material and Methods

#### Appendix A.1.1. Model

We framed the task as a dense classification problem, whereby each pixel in the image was determined to either contain a radiolucent periapical alteration (positive) or not (negative). We used a model architecture based on U-Net [37] with same padding convolutional layers, five levels of resolution and batch normalization layers [42] introduced prior to each activation layer (Table A1). The use of this architecture was inspired by the outcome of an open innovation competition, held on TopCoder.com, Wipro, Bengaluru, India. The network accepted as input 256 × 512 pixel pre-processed panoramic radiographic images in (0,1)^256 × 512^, and output an intensity map in (0,1)^256 × 512^, where pixel intensities near unity indicated regions with a high confidence of being positive. The preprocessing involved resizing the original radiographic images, which varied in shape, to a reference shape (1280 × 2560 pixels), followed by cropping of the image boundaries (100 pixels on the upper and lower boundaries, and 300 pixels on the left and right boundaries), and finally resizing the cropped images to a standard target shape of 256 × 512 pixels. The resizing was performed using bi-linear interpolation. The pixel intensities of the images were subsequently scaled to the interval (0,1).

The model was trained on 7/8 of the training data set (the remaining 1/8 was used for validation, e.g., to check for overfitting) using an objective function based on the dice loss function [43] with data augmentation, including random translations in the horizontal and vertical directions (up to ±20 pixels in each direction), horizontal flips and rotations (up to ±15 degrees). Images were augmented with a probability of one half upon each visit during training. The model was trained using Adam optimization [44] for 25 epochs and in mini-batches of 10 images. The initial learning rate was 0.001 and the learning rate was subsequently reduced using exponential decay with a decay constant 0.1/epoch. The model hyperparameters were selected on the basis of best performance on the validation data set. The model training was performed using an Nvidia Tesla K80 GPU accelerator.

An ensemble model was subsequently created by combining the predictions of 10 trained models, where each model was trained using the same selected hyperparameters and training period as previously described. Each constituent model, however, was trained on 10 choose 9 disjoint subsets of the training data set, randomly split at the patient level. An ensemble intensity map was finally produced by taking the mean output produced by the 10 constituent models.

**Table A1:** U-Net architecture.

Layer	Type	Kernel Size	Number of Kernels	Input Dimensions	Activation Function
1	Conv2D	3 × 3	64	256 × 512	ReLU
2	Conv2D	3 × 3	64	256 × 512	ReLU
3	MaxPool	2 × 2	-	256 × 512	-
4	Conv2D	3 × 3	128	128 × 256	ReLU
5	Conv2D	3 × 3	128	128 × 256	ReLU
6	MaxPool	2 × 2	-	128 × 256	-
7	Conv2D	3 × 3	256	64 × 128	ReLU
8	Conv2D	3 × 3	256	64 × 128	ReLU
9	MaxPool	2 × 2	-	64 × 128	-
10	Conv2D	3 × 3	512	32 × 64	ReLU
11	Conv2D	3 × 3	512	32 × 64	ReLU
12	MaxPool	2 × 2	-	32 × 64	-
13	Conv2D	3 × 3	1024	16 × 32	ReLU
14	Conv2D	3 × 3	1024	16 × 32	ReLU
15	Dropout	-	-	16 × 32	-
16	UpConv2D	2 × 2	512	16 × 32	-
-	Concat(11)	-	-	-	-
17	Conv2D	3 × 3	512	32 × 64	ReLU
18	Conv2D	3 × 3	512	32 × 64	ReLU
19	UpConv2D	2 × 2	256	32 × 64	-
-	Concat(8)	-	-	-	-
20	Conv2D	3 × 3	256	64 × 128	ReLU
21	Conv2D	3 × 3	256	64 × 128	ReLU
22	UpConv2D	2 × 2	128	64 × 128	-
-	Concat(5)	-	-	-	-
23	Conv2D	3 × 3	128	128 × 256	ReLU
24	Conv2D	3 × 3	128	128 × 256	ReLU
25	UpConv2D	2 × 2	64	128 × 256	-
-	Concat(3)	-	-	-	-
26	Conv2D	3 × 3	64	256 × 512	ReLU
27	Conv2D	3 × 3	64	256 × 512	ReLU
28	Conv2D	1 × 1	1	256 × 512	Sigmoid

Legend: Conv2D: two-dimensional convolutional layer; MaxPool: max-pooling layer; UpConv2D: two-dimensional transpose-convolutional layer; Concat(k): concatenate channels with output of layer k.

#### Appendix A.1.2. Inference and Post-Processing

The output of our ensemble model was postprocessed prior to evaluation (Figure A1, Figure A2, Figure A3 and Figure A4). A Gaussian filter (width of three pixels) was first applied to the 256 × 512 pixel output intensity map to smooth out any ultra-local variations; subsequently a peak finding algorithm was used to determine all local maxima and associated confidence scores. The latter were defined as the value of the intensity map at each local maximum. The peak finding algorithm involved the application of a maximum filter to the intensity map with a neighborhood radius of four pixels and subsequent identification of points (peaks) where the filtered image equaled the original image. The maximum filter neighborhood radius was selected based on the observation that no more than 0.5% of the images in the preprocessed training data set had a minimum centroid distance between lesions less than four pixels.

#### Appendix A.1.3. Evaluation Metrics

The performance of our algorithm was assessed in terms of PPV, TPR, F_1_ score and AP, which in turn depends on true positive (N_TP_), false positive (N_FP_) and false negative (N_FN_) counts as a function of the threshold. Predictions at a given threshold were defined as the subset of peak locations detected in the output intensity map which had confidence scores greater than the specified threshold. For a given set of predictions, N_TP_ was defined as the total number of positive regions that were within an error tolerance determined as the lessor of either four pixels (a conservative choice based on the typical inter-root distance; see Figure A5) or half the minimum distance between contours within the image if multiple lesions were present (note that with this definition, neighboring error regions can never overlap). The introduction of such an error tolerance was necessary to account for the inherent variability in the interpretation of the region of interest by the OMF surgeon who produced the labels, noting that the ground truth contours were tightly drawn. N_FN_ was defined as the number of positive regions of interest with no candidate points located within the specified error tolerance. N_FP_ was defined as the total number of candidate points that were located at a distance greater than the specified error tolerance from any region of interest.
